# Supplementary material for: A Likelihood Approach for Real-Time Calibration of Stochastic Compartmental Epidemic Models
Source: PLoS Comput Biol. 2017 Jan 17;13(1):e1005257. doi: 10.1371/journal.pcbi.1005257 (PMC5240920; doi:10.1371/journal.pcbi.1005257)
Supplement: S1 File — (TAR.GZ) [file pcbi.1005257.s014.tar.gz › HSPH_Online-SI-Revision/MSS10/n1-extreme/n1-extreme_NEW2_table4.pdf]

1	newly 3 weeks spec	16.	16.49	{11., 22.}	20.9375	0.0923699	newly 3 weeks cumul	86.	78.12	{69., 87.}	10.1163	0.0473523	newly 5 weeks spec	10.	6.44	{3., 10.}	39.2	0.253923	newly 5 weeks cumul	112.	94.38	{86., 104.}	15.7321	0.075656	newly 56 weeks cumul	131.	105.34	{93., 116.}	19.5878	0.0960103
2	newly 3 weeks spec	15.	13.04	{8., 17.}	21.2	0.110597	newly 3 weeks cumul	66.	53.7	{46., 61.}	18.697	0.0922003	newly 5 weeks spec	14.	6.94	{3., 11.}	50.4286	0.348832	newly 5 weeks cumul	87.	70.03	{61., 80.}	19.5287	0.0961813	newly 52 weeks cumul	117.	89.77	{77., 105.}	23.2735	0.118358
3	newly 3 weeks spec	43.	27.84	{20., 36.}	35.2558	0.199152	newly 3 weeks cumul	138.	115.06	{101., 128.}	17.1739	0.0830882	newly 5 weeks spec	26.	14.65	{8., 21.}	44.3462	0.281338	newly 5 weeks cumul	184.	150.76	{132., 172.}	18.5543	0.0907836	newly 54 weeks cumul	247.	192.37	{157., 235.}	22.2146	0.113712
4	newly 3 weeks spec	35.	24.02	{14., 36.}	35.9429	0.210964	newly 3 weeks cumul	105.	96.96	{66., 131.}	22.381	0.106426	newly 5 weeks spec	26.	13.36	{5., 23.}	48.6154	0.341744	newly 5 weeks cumul	155.	128.96	{87., 175.}	24.2323	0.126244	newly 53 weeks cumul	252.	167.85	{108., 237.}	34.2579	0.201616
5	newly 3 weeks spec	18.	2.38	{0., 6.}	86.7778	1.22995	newly 3 weeks cumul	78.	13.86	{6., 22.}	82.2308	0.803769	newly 5 weeks spec	14.	0.63	{0., 2.}	95.5	1.74194	newly 5 weeks cumul	116.	15.77	{6., 26.}	86.4052	0.923201	newly 46 weeks cumul	161.	16.62	{6., 27.}	89.677	1.05049
6	newly 3 weeks spec	29.	5.49	{0., 14.}	81.2759	1.21513	newly 3 weeks cumul	76.	38.96	{9., 72.}	52.7895	0.4124	newly 5 weeks spec	16.	0.86	{0., 3.}	94.625	1.84608	newly 5 weeks cumul	103.	42.13	{9., 80.}	59.9903	0.506944	newly 51 weeks cumul	158.	42.97	{9., 81.}	72.8038	0.682803
7	newly 3 weeks spec	23.	20.33	{14., 28.}	21.6087	0.106923	newly 3 weeks cumul	107.	79.92	{68., 90.}	26.1495	0.133908	newly 5 weeks spec	18.	11.79	{6., 18.}	37.1667	0.248484	newly 5 weeks cumul	144.	108.07	{91., 124.}	26.1458	0.133474	newly 55 weeks cumul	190.	142.7	{118., 164.}	25.8842	0.132459
8	newly 3 weeks spec	21.	29.1	{23., 34.}	39.0476	0.138256	newly 3 weeks cumul	65.	117.25	{107., 126.}	80.3846	0.255505	newly 5 weeks spec	12.	15.14	{10., 20.}	32.3333	0.116928	newly 5 weeks cumul	85.	153.46	{146., 161.}	80.5412	0.256213	newly 49 weeks cumul	108.	196.97	{188., 207.}	82.3796	0.26068
9	newly 3 weeks spec	14.	20.28	{13., 30.}	49.2857	0.162077	newly 3 weeks cumul	73.	89.86	{74., 105.}	24.2192	0.0873064	newly 5 weeks spec	7.	10.43	{5., 17.}	63.	0.200422	newly 5 weeks cumul	94.	114.52	{93., 137.}	23.1064	0.0832636	newly 56 weeks cumul	112.	136.35	{107., 164.}	23.7589	0.084431
10	newly 3 weeks spec	36.	30.24	{23., 37.}	18.1667	0.0919094	newly 3 weeks cumul	109.	118.91	{107., 130.}	9.99083	0.0407161	newly 5 weeks spec	14.	17.4	{13., 22.}	28.	0.102594	newly 5 weeks cumul	147.	159.98	{148., 169.}	9.2381	0.0379626	newly 49 weeks cumul	210.	216.2	{201., 230.}	4.87619	0.0206794
11	newly 3 weeks spec	14.	17.82	{11., 25.}	37.4286	0.135809	newly 3 weeks cumul	74.	77.96	{62., 93.}	13.9189	0.059655	newly 5 weeks spec	6.	9.12	{4., 14.}	66.3333	0.232852	newly 5 weeks cumul	95.	99.8	{77., 122.}	14.8842	0.0643391	newly 55 weeks cumul	108.	119.06	{91., 143.}	19.5741	0.0802964
12	newly 3 weeks spec	26.	13.37	{5., 23.}	50.7308	0.396291	newly 3 weeks cumul	91.	69.15	{38., 94.}	28.6044	0.178487	newly 5 weeks spec	9.	4.81	{0., 12.}	58.3333	0.650779	newly 5 weeks cumul	114.	82.51	{50., 116.}	31.4123	0.199184	newly 56 weeks cumul	127.	89.8	{50., 126.}	33.5433	0.215658
13	newly 3 weeks spec	35.	11.09	{3., 20.}	68.3143	0.623933	newly 3 weeks cumul	94.	45.61	{17., 71.}	51.4787	0.369531	newly 5 weeks spec	33.	6.62	{0., 16.}	79.9394	0.952898	newly 5 weeks cumul	163.	60.51	{25., 100.}	62.8773	0.49522	newly 49 weeks cumul	326.	75.34	{25., 122.}	76.8896	0.714271
14	newly 3 weeks spec	7.	11.36	{7., 16.}	65.4286	0.204709	newly 3 weeks cumul	41.	49.88	{42., 57.}	22.8293	0.0872724	newly 5 weeks spec	2.	5.65	{2., 9.}	186.5	0.41887	newly 5 weeks cumul	46.	62.96	{54., 72.}	36.9565	0.133956	newly 53 weeks cumul	53.	74.15	{62., 89.}	40.2453	0.142997
15	newly 3 weeks spec	12.	25.26	{19., 31.}	110.5	0.315118	newly 3 weeks cumul	65.	88.78	{79., 100.}	36.5846	0.133752	newly 5 weeks spec	12.	16.44	{11., 21.}	41.	0.142949	newly 5 weeks cumul	89.	126.03	{114., 138.}	41.6067	0.149934	newly 48 weeks cumul	106.	198.9	{178., 218.}	87.6415	0.271957
16	newly 3 weeks spec	31.	10.75	{4., 20.}	65.3226	0.562294	newly 3 weeks cumul	81.	48.66	{23., 81.}	43.3086	0.279562	newly 5 weeks spec	20.	5.62	{0., 12.}	71.9	0.801201	newly 5 weeks cumul	118.	61.73	{32., 106.}	48.4831	0.330168	newly 55 weeks cumul	180.	72.22	{34., 120.}	59.8778	0.449642
17	newly 3 weeks spec	33.	21.8	{15., 30.}	35.6364	0.207023	newly 3 weeks cumul	111.	87.9	{72., 103.}	21.6396	0.110222	newly 5 weeks spec	26.	10.97	{5., 18.}	57.8077	0.417098	newly 5 weeks cumul	160.	115.16	{90., 136.}	28.625	0.151687	newly 52 weeks cumul	266.	142.81	{110., 170.}	46.312	0.278083
18	newly 3 weeks spec	26.	31.22	{25., 37.}	22.3846	0.0849895	newly 3 weeks cumul	118.	124.24	{114., 134.}	6.91525	0.0287967	newly 5 weeks spec	13.	17.3	{12., 22.}	38.7692	0.138518	newly 5 weeks cumul	153.	164.13	{153., 173.}	7.64052	0.0316769	newly 55 weeks cumul	200.	214.24	{202., 225.}	7.2	0.0298896
19	newly 3 weeks spec	32.	28.95	{21., 36.}	16.7813	0.0820104	newly 3 weeks cumul	144.	113.48	{103., 124.}	21.1944	0.104619	newly 5 weeks spec	11.	16.47	{11., 22.}	53.3636	0.178441	newly 5 weeks cumul	177.	152.04	{139., 164.}	14.1017	0.0667867	newly 56 weeks cumul	231.	201.14	{183., 220.}	13.0823	0.0619036
20	newly 3 weeks spec	8.	2.92	{0., 10.}	76.	0.923865	newly 3 weeks cumul	57.	14.12	{3., 49.}	75.5088	0.8537	newly 5 weeks spec	5.	1.35	{0., 4.}	78.6	1.07613	newly 5 weeks cumul	69.	17.57	{4., 60.}	74.8551	0.835704	newly 49 weeks cumul	94.	21.62	{4., 71.}	77.3617	0.879468
21	newly 3 weeks spec	40.	37.2	{31., 44.}	12.	0.0561842	newly 3 weeks cumul	123.	123.75	{113., 134.}	5.09756	0.0220561	newly 5 weeks spec	31.	28.62	{22., 35.}	13.9355	0.0661037	newly 5 weeks cumul	179.	184.95	{170., 200.}	5.72626	0.0241348	newly 52 weeks cumul	267.	321.67	{293., 351.}	20.4757	0.0798429
22	newly 3 weeks spec	22.	12.03	{8., 17.}	45.5	0.283024	newly 3 weeks cumul	81.	51.86	{42., 62.}	35.9753	0.197949	newly 5 weeks spec	11.	6.71	{3., 11.}	41.	0.269214	newly 5 weeks cumul	100.	67.67	{56., 78.}	32.33	0.173556	newly 53 weeks cumul	148.	86.11	{67., 104.}	41.8176	0.240725
23	newly 3 weeks spec	17.	26.27	{18., 34.}	55.1176	0.181356	newly 3 weeks cumul	88.	102.43	{88., 115.}	17.3068	0.0679033	newly 5 weeks spec	9.	15.23	{9., 22.}	71.2222	0.216285	newly 5 weeks cumul	105.	137.36	{120., 156.}	30.819	0.114466	newly 55 weeks cumul	155.	179.21	{150., 205.}	17.0387	0.0667441
24	newly 3 weeks spec	14.	13.	{7., 20.}	27.4286	0.137557	newly 3 weeks cumul	65.	62.35	{44., 75.}	13.5231	0.0659431	newly 5 weeks spec	7.	5.55	{2., 9.}	35.5714	0.25977	newly 5 weeks cumul	79.	76.35	{53., 93.}	14.7468	0.0716369	newly 56 weeks cumul	97.	87.07	{59., 106.}	15.268	0.0794215
25	newly 3 weeks spec	37.	28.63	{19., 37.}	25.2703	0.134982	newly 3 weeks cumul	118.	117.17	{97., 135.}	9.82203	0.0428716	newly 5 weeks spec	14.	15.43	{9., 23.}	29.9286	0.125453	newly 5 weeks cumul	153.	153.84	{128., 179.}	10.4183	0.0445247	newly 53 weeks cumul	253.	191.13	{159., 225.}	25.751	0.132517
26	newly 3 weeks spec	7.	12.96	{9., 16.}	85.7143	0.257767	newly 3 weeks cumul	46.	56.09	{50., 63.}	22.1522	0.0853026	newly 5 weeks spec	5.	6.46	{3., 10.}	47.6	0.176349	newly 5 weeks cumul	56.	71.09	{65., 78.}	27.1607	0.103285	newly 55 weeks cumul	70.	85.69	{78., 93.}	22.6714	0.0878817
27	newly 3 weeks spec	41.	21.24	{14., 29.}	48.1951	0.302187	newly 3 weeks cumul	120.	79.22	{65., 94.}	33.9833	0.187904	newly 5 weeks spec	16.	13.95	{8., 19.}	26.0625	0.14088	newly 5 weeks cumul	166.	109.57	{95., 127.}	33.994	0.188125	newly 50 weeks cumul	260.	157.51	{127., 183.}	39.4192	0.225737
28	newly 3 weeks spec	13.	23.7	{17., 30.}	82.7692	0.253323	newly 3 weeks cumul	77.	92.71	{84., 102.}	21.1039	0.0822253	newly 5 weeks spec	17.	13.53	{8., 18.}	26.0588	0.139839	newly 5 weeks cumul	109.	123.19	{115., 132.}	13.9358	0.0565519	newly 54 weeks cumul	162.	162.67	{154., 173.}	4.12963	0.018532
29	newly 3 weeks spec	7.	1.71	{0., 4.}	75.5714	0.926939	newly 3 weeks cumul	71.	11.55	{5., 21.}	83.7324	0.859249	newly 5 weeks spec	8.	0.46	{0., 2.}	94.25	1.57123	newly 5 weeks cumul	91.	12.76	{5., 23.}	85.978	0.929218	newly 51 weeks cumul	100.	13.17	{5., 24.}	86.83	0.960963
30	newly 3 weeks spec	10.	15.01	{10., 19.}	53.7	0.178956	newly 3 weeks cumul	42.	63.	{55., 71.}	50.1905	0.173919	newly 5 weeks spec	6.	8.75	{3., 13.}	60.5	0.209741	newly 5 weeks cumul	53.	83.79	{69., 96.}	58.2075	0.195736	newly 46 weeks cumul	78.	107.94	{81., 127.}	39.641	0.140825
31	newly 3 weeks spec	10.	21.48	{13., 28.}	114.8	0.316597	newly 3 weeks cumul	87.	88.86	{71., 103.}	11.5172	0.0508183	newly 5 weeks spec	4.	10.69	{5., 16.}	169.25	0.400533	newly 5 weeks cumul	99.	114.56	{95., 135.}	19.8182	0.079054	newly 55 weeks cumul	122.	143.25	{118., 165.}	21.4672	0.0855649
32	newly 3 weeks spec	6.	20.27	{14., 27.}	237.833	0.517138	newly 3 weeks cumul	62.	85.91	{75., 98.}	38.5645	0.139864	newly 5 weeks spec	6.	10.51	{6., 16.}	81.1667	0.247611	newly 5 weeks cumul	78.	110.87	{97., 123.}	42.141	0.151081	newly 55 weeks cumul	82.	137.22	{118., 157.}	67.3415	0.221523
33	newly 3 weeks spec	35.	35.82	{28., 44.}	13.6	0.0585356	newly 3 weeks cumul	123.	133.5	{120., 146.}	10.0163	0.0410975	newly 5 weeks spec	29.	23.66	{17., 31.}	21.1724	0.109371	newly 5 weeks cumul	183.	186.57	{173., 200.}	5.02186	0.0214298	newly 51 weeks cumul	278.	274.92	{255., 294.}	4.66906	0.020683
34	newly 3 weeks spec	27.	17.27	{11., 25.}	37.1481	0.219675	newly 3 weeks cumul	98.	76.46	{56., 89.}	22.5306	0.116089	newly 5 weeks spec	12.	8.08	{3., 12.}	37.6667	0.252585	newly 5 weeks cumul	137.	96.21	{72., 116.}	30.2993	0.163051	newly 54 weeks cumul	165.	114.33	{84., 139.}	31.7636	0.173221
35	newly 3 weeks spec	25.	23.48	{17., 31.}	18.64	0.0870105	newly 3 weeks cumul	103.	95.05	{84., 107.}	9.50485	0.0440467	newly 5 weeks spec	20.	13.	{7., 19.}	35.8	0.216364	newly 5 weeks cumul	144.	125.59	{113., 139.}	12.8681	0.0612064	newly 55 weeks cumul	192.	161.76	{143., 178.}	15.8646	0.0767205
36	newly 3 weeks spec	28.	25.05	{19., 31.}	16.9643	0.0832713	newly 3 weeks cumul	103.	99.98	{89., 111.}	6.73786	0.0301093	newly 5 weeks spec	9.	14.32	{9., 19.}	63.1111	0.201362	newly 5 weeks cumul	131.	133.45	{121., 146.}	5.94656	0.0254168	newly 52 weeks cumul	175.	174.21	{156., 190.}	6.30286	0.0275786
37	newly 3 weeks spec	25.	6.89	{3., 10.}	72.44	0.608897	newly 3 weeks cumul	95.	27.63	{21., 34.}	70.9158	0.553723	newly 5 weeks spec	12.	4.26	{1., 8.}	64.5	0.547602	newly 5 weeks cumul	128.	37.31	{29., 45.}	70.8516	0.554522	newly 47 weeks cumul	171.	51.62	{37., 66.}	69.8129	0.544723
38	newly 3 weeks spec	18.	21.45	{16., 26.}	24.3889	0.0933245	newly 3 weeks cumul	76.	89.7	{81., 98.}	18.4737	0.0727846	newly 5 weeks spec	9.	11.68	{7., 16.}	39.1111	0.139792	newly 5 weeks cumul	96.	117.08	{108., 125.}	22.3542	0.0871351	newly 52 weeks cumul	127.	146.55	{136., 158.}	15.9606	0.0639016
39	newly 3 weeks spec	23.	31.5	{25., 39.}	37.7391	0.134362	newly 3 weeks cumul	103.	125.37	{114., 135.}	21.7184	0.084515	newly 5 weeks spec	6.	16.82	{11., 23.}	180.333	0.430812	newly 5 weeks cumul	136.	164.07	{155., 175.}	20.6397	0.0809923	newly 55 weeks cumul	164.	209.73	{198., 219.}	27.8841	0.106452
40	newly 3 weeks spec	14.	24.41	{17., 30.}	74.6429	0.231566	newly 3 weeks cumul	66.	92.72	{85., 102.}	40.4848	0.146428	newly 5 weeks spec	15.	14.74	{9., 21.}	24.4	0.114079	newly 5 weeks cumul	96.	126.45	{115., 140.}	31.7188	0.118497	newly 53 weeks cumul	143.	178.08	{158., 194.}	24.5315	0.0938482
41	newly 3 weeks spec	19.	26.77	{19., 35.}	44.5789	0.153712	newly 3 weeks cumul	71.	106.12	{89., 122.}	49.4648	0.170055	newly 5 weeks spec	8.	14.74	{8., 21.}	87.	0.250998	newly 5 weeks cumul	101.	140.76	{119., 168.}	39.3663	0.139107	newly 48 weeks cumul	154.	178.43	{149., 203.}	18.0455	0.0675531
42	newly 3 weeks spec	21.	24.62	{18., 31.}	24.	0.0920108	newly 3 weeks cumul	118.	98.89	{90., 107.}	16.2288	0.0779077	newly 5 weeks spec	14.	14.	{9., 20.}	22.8571	0.102083	newly 5 weeks cumul	148.	131.	{121., 140.}	11.4865	0.053629	newly 56 weeks cumul	159.	172.09	{160., 185.}	8.4717	0.034832
43	newly 3 weeks spec	41.	21.64	{15., 28.}	48.1463	0.301514	newly 3 weeks cumul	137.	89.11	{75., 101.}	35.5401	0.193653	newly 5 weeks spec	20.	10.75	{5., 16.}	46.85	0.317534	newly 5 weeks cumul	181.	115.04	{99., 134.}	36.895	0.203665	newly 55 weeks cumul	229.	141.97	{115., 163.}	38.6681	0.217072
44	newly 3 weeks spec	21.	22.81	{16., 30.}	21.8571	0.0904315	newly 3 weeks cumul	78.	93.69	{81., 107.}	20.3718	0.0783894	newly 5 weeks spec	11.	12.06	{7., 17.}	28.5455	0.119032	newly 5 weeks cumul	103.	121.69	{105., 136.}	18.6699	0.0727087	newly 53 weeks cumul	147.	151.87	{133., 170.}	7.95238	0.0336346
45	newly 3 weeks spec	42.	27.47	{20., 34.}	34.5952	0.195274	newly 3 weeks cumul	127.	114.73	{92., 130.}	11.2835	0.0545693	newly 5 weeks spec	16.	13.43	{7., 19.}	24.4375	0.143586	newly 5 weeks cumul	166.	146.55	{112., 167.}	12.5843	0.0625353	newly 56 weeks cumul	228.	183.52	{132., 213.}	19.5088	0.101192
46	newly 3 weeks spec	28.	21.57	{15., 29.}	26.6071	0.14227	newly 3 weeks cumul	111.	89.74	{79., 101.}	19.4234	0.0958035	newly 5 weeks spec	15.	11.	{6., 17.}	31.2	0.181224	newly 5 weeks cumul	142.	115.6	{98., 129.}	18.6197	0.0916195	newly 53 weeks cumul	196.	141.86	{121., 158.}	27.6224	0.142541
47	newly 3 weeks spec	37.	24.2	{16., 32.}	34.8108	0.201437	newly 3 weeks cumul	141.	96.53	{81., 111.}	31.539	0.168043	newly 5 weeks spec	23.	13.24	{7., 19.}	42.7826	0.275034	newly 5 weeks cumul	188.	126.65	{105., 151.}	32.633	0.175195	newly 54 weeks cumul	283.	159.29	{125., 194.}	43.7138	0.255068
48	newly 3 weeks spec	34.	20.2	{9., 35.}	43.8824	0.297945	newly 3 weeks cumul	113.	79.65	{46., 127.}	35.2124	0.208122	newly 5 weeks spec	18.	10.8	{4., 19.}	46.3333	0.350271	newly 5 weeks cumul	163.	105.9	{59., 172.}	37.8896	0.235487	newly 54 weeks cumul	230.	137.13	{62., 230.}	41.6391	0.275108
49	newly 3 weeks spec	30.	21.39	{14., 28.}	30.3	0.171358	newly 3 weeks cumul	104.	91.24	{72., 104.}	13.1154	0.0647238	newly 5 weeks spec	16.	11.53	{6., 16.}	30.4375	0.185697	newly 5 weeks cumul	140.	117.97	{95., 136.}	15.8643	0.0795468	newly 56 weeks cumul	177.	147.07	{111., 168.}	16.9096	0.0861846
50	newly 3 weeks spec	29.	18.	{12., 23.}	37.931	0.219161	newly 3 weeks cumul	85.	62.03	{52., 73.}	27.0235	0.139714	newly 5 weeks spec	17.	14.03	{9., 19.}	25.4706	0.133472	newly 5 weeks cumul	130.	91.92	{82., 104.}	29.2923	0.152383	newly 54 weeks cumul	176.	166.98	{142., 197.}	11.0795	0.050502
